# The Evidence for Effective Inhibition of *I*_Na_ Produced by Mirogabalin ((1R,5S,6S)-6-(aminomethyl)-3-ethyl-bicyclo [3.2.0] hept-3-ene-6-acetic acid), a Known Blocker of Ca_V_ Channels

**DOI:** 10.3390/ijms23073845

**Published:** 2022-03-31

**Authors:** Chao-Liang Wu, Chao-Wei Chuang, Hsin-Yen Cho, Tzu-Hsien Chuang, Sheng-Nan Wu

**Affiliations:** 1 Department of Medical Research, Ditmanson Medical Foundation Chia-Yi Christian Hospital, Chiayi City 60002, Taiwan; wumolbio@mail.ncku.edu.tw; 2 Department of Ophthalmology, Ditmanson Medical Foundation Chia-Yi Christian Hospital, Chiayi City 60002, Taiwan; 07006@cych.org.tw; 3 Department of Physiology, National Cheng Kung University Medical College, Tainan City 70101, Taiwan; s36094083@gs.ncku.edu.tw (H.-Y.C.); s36091051@gs.ncku.edu.tw (T.-H.C.); 4 Institute of Basic Medical Sciences, National Cheng Kung University Medical College, Tainan City 70101, Taiwan

**Keywords:** mirogabalin (Tarlige^®^, 1R,5S,6S)-6-(aminomethyl)-3-ethyl-bicyclo [3.2.0] hept-3-ene-6-acetic acid), voltage-gated Na^+^ current, window Na^+^ current, resurgent Na^+^ current, persistent Na^+^ current, hysteresis, current kinetics, pulse train stimulation

## Abstract

Mirogabalin (MGB, Tarlige^®^), an inhibitor of the α_2_δ-1 subunit of voltage-gated Ca^2+^ (Ca_V_) channels, is used as a way to alleviate peripheral neuropathic pain and diabetic neuropathy. However, to what extent MGB modifies the magnitude, gating, and/or hysteresis of various types of plasmalemmal ionic currents remains largely unexplored. In pituitary tumor (GH_3_) cells, we found that MGB was effective at suppressing the peak (transient, *I*_Na(T)_) and sustained (late, *I*_Na(L)_) components of the voltage-gated Na^+^ current (*I*_Na_) in a concentration-dependent manner, with an effective IC_50_ of 19.5 and 7.3 μM, respectively, while the *K*_D_ value calculated on the basis of minimum reaction scheme was 8.2 μM. The recovery of *I*_Na(T)_ inactivation slowed in the presence of MGB, although the overall current–voltage relation of *I*_Na(T)_ was unaltered; however, there was a leftward shift in the inactivation curve of the current. The magnitude of the window (*I*_Na(W)_) or resurgent *I*_Na_ (*I*_Na(R)_) evoked by the respective ascending or descending ramp pulse (V_ramp_) was reduced during cell exposure to MGB. MGB-induced attenuation in *I*_Na(W)_ or *I*_Na(R)_ was reversed by the further addition of tefluthrin, a pyrethroid insecticide known to stimulate *I*_Na_. MGB also effectively lessened the strength of voltage-dependent hysteresis of persistent *I*_Na_ in response to the isosceles triangular V_ramp_. The cumulative inhibition of *I*_Na(T),_ evoked by pulse train stimulation, was enhanced in its presence. Taken together, in addition to the inhibition of Ca_V_ channels, the Na_V_ channel attenuation produced by MGB might have an impact in its analgesic effects occurring in vivo.

## 1. Introduction

Mirogabalin (MGB, DS-5565, Tarlige^®^, (1R,5S,6S)-6-(aminomethyl)-3-ethyl-bicyclo [3.2.0]hept-3-ene-6-acetic acid), an orally administered gabapentinoid, is a novel, preferentially selective ligand for the α_2_δ-1 subunit of voltage-gated Ca^2+^ (Ca_V_) channels, and has been used in trials for investigations in the treatment of post-herpetic neuralgia, pain associated with fibromyalgia, and diabetic peripheral neuropathic pain [1,2,3,4,5,6,7,8,9,10,11,12,13,14,15,16]. Owing to the considerable research effort, this compound is being investigated for the treatment of peripheral neuropathic pain and fibromyalgia and has demonstrated promising results in patients with diabetic peripheral neuropathy [2,3,4,5,6,9,11,14,15]. Moreover, it has recently been shown to be effective in alleviating anxiety, and in improving cognitive impairments in rats injected repeatedly and intramuscularly with acidic saline [12,13,17]. Based mostly on the potent and long-lasting analgesic effects of MGB, the α_2_δ-1 subunit of Ca_V_1- and Ca_V_2-type voltage-gated Ca^2+^ (Ca_V_) channels is considered to play a role in the occurrence of neuropathic pain [3,4,5,7,14,18,19,20]. However, to the best of our knowledge, it is not yet clear whether the presence of MGB has any effects on other types of membrane ionic currents.

The voltage-gated Na^+^ (Na_V_) channels are recognized to play a pivotal role in the generation and propagation of action potentials in excitable membranes. The Na_V_ channel protein contains four homologous domains (D1–D4), each with six transmembrane segments (S1–S6). When rapid depolarization is established, Na_V_ channels readily go through rapid transitions from the closed (resting) state to the open state and then swiftly to the inactivated state. Genetic defects (i.e., gain-of-function) in Na_V_ channel inactivation that led to small, sustained Na^+^ currents (*I*_Na_) (i.e., late Na^+^ current [*I*_Na(L)_]) following the occurrence of action potential firing are recognized to have devastating consequences, including neuropathic pain [21,22,23,24].

Nine pore-forming α-subunits (Na_V_1.1–1.9) are distributed among excitable mammalian tissues, including central and peripheral nervous systems, and endocrine or heart tissue [25]. The Na_V_1.7 and Na_V_1.8 subtypes have emerged as key molecules involved in peripheral pain processing and in the development of an increased pain sensitivity associated with inflammation and tissue injury [26,27,28,29,30]. Several activators or inhibitors have been increasingly reported to preferentially modify the late component of the voltage-gated Na^+^ current (i.e., *I*_Na(L)_) [24,31,32,33,34,35,36,37]. However, to date, the issue of whether or how MGB could perturb the magnitude, kinetic gating, or hysteresis of membrane ionic currents (e.g., *I*_Na_) is poorly characterized.

Therefore, in this study, we intended to determine the possible underlying mechanism of MGB actions on the perturbation on different ionic currents (e.g., *I*_Na_) residing in excitable cells (e.g., pituitary GH_3_ lactotrophs). The present investigations obtained in this study highlight the evidence showing that MGB can differentially inhibit the transient (*I*_Na(T)_) and late (*I*_Na(L)_) components of *I*_Na_ in a concentration-dependent manner in these cells. In addition to the inhibition of Ca_V_ channels, MGB-mediated interference within the activity of the Na_V_ channels will converge to act use-dependently on the magnitude, gating, and hysteresis of *I*_Na_ in different types of excitable cells.

## 2. Results

### 2.1. Inhibitory Effect of MGB on Voltage-Gated Na^+^ Current (I_Na_) Measured from Pituitary GH_3_ Cells

**Figure 1:**
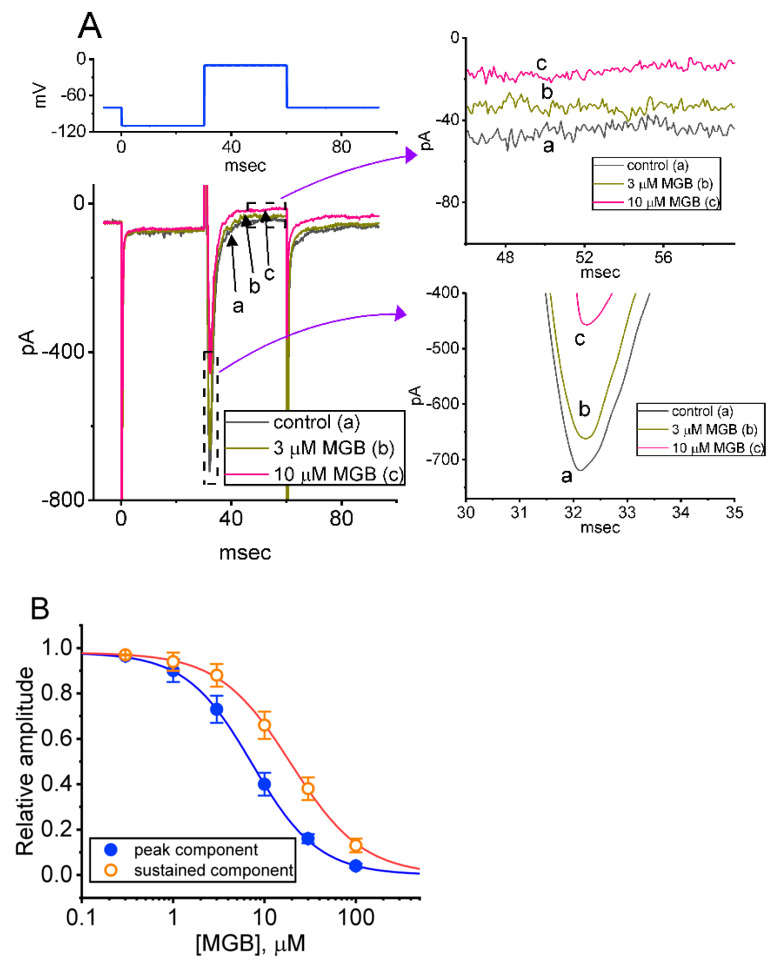
Effect of mirogabalin (MGB) on voltage-gated Na^+^ current (*I*_Na_) identified in pituitary GH_3_ cells. In this series of experiments, we bathed cells in Ca^2+^-free Tyrode’s solution, which contained 10 mM tetraethylammonium chloride (TEA) and 0.5 mM CdCl_2_, and the electrode that was used was filled up with a solution containing Cs^+^. (**A**) Representative current traces acquired in the control period (a) (i.e., absence of MGB) and during the exposure to 3 μM MGB (b) or 10 μM MGB (c). The voltage clamp protocol that we applied is illustrated in the upper part. The graphs shown in the right side of (**A**) indicate the expanded records from the left side (dashed boxes). (**B**) Concentration–response curve of MGB-induced block of peak (transient) *I*_Na_ (*I*_Na(T)_) or sustained (late) *I*_Na_ (*I*_Na(L)_) occurring in GH_3_ cells. The continuous line drawn represents the goodness-of-fit to the modified Hill equation, as described in Section 4. The IC_50_ values for the MGB-induced inhibition of *I*_Na(T)_ and *I*_Na(L)_ were optimally estimated to be 19.5 and 7.3 μM, respectively. Each point represents the mean ± SEM (n = 8–10).

For the first stage of measurements, we kept cells bathed in Ca^2+^-free Tyrode’s solution, which contained 10 mM tetraethylammonium chloride (TEA) and 0.5 mM CdCl_2_, and the electrode used was filled up with a Cs^+^-containing solution. TEA and CdCl_2_ were used to block most of the K^+^ and Ca^2+^ currents, respectively. In this set of whole-cell current recordings, the tested cell was held at −80 mV, a hyperpolarizing pulse of −100 mV was then applied for 30 ms to precede the depolarizing command voltage from −100 to −10 mV, and such a depolarizing step was then imposed to evoke *I*_Na_. Under this experimental protocol, we were able to detect the emergence of an inward current (i.e., inward flux of cations) which displayed the rapidly activating and inactivating time course (Figure 1A). In response to a brief rectangular pulse, this type of transient inward current was sensitive to inhibition or stimulation by tetrodotoxin (TTX, 1 μM) or tefluthrin (Tef, 10 μM), respectively; it has hence been identified as a TTX-sensitive voltage-gated Na^+^ current (*I*_Na_) [24,32,34,35,36,38]. It is of note that one minute after cells were exposed to MGB, the amplitude of peak *I*_Na_ (or transient *I*_Na_, [*I*_Na(T)_]) progressively decreased in combination with a concomitant increase in the inactivation time course of the current. For example, the addition of MGB (10 μM) markedly decreased *I*_Na(T)_ from 729 ± 33 to 442 ± 23 pA (n = 9, *p* < 0.05). Additionally, the time constant in the slow component of the current inactivation (τ_inact(S)_) was concurrently shortened to 2.2 ± 0.2 ms (n = 9, *p* < 0.05) from a control of 3.9 ±0.3 ms (n = 9). However, no obvious difference in the fast component of the current inactivation was demonstrated in the presence of MGB. After the washout of the compound, current amplitude returned to 719 ± 29 pA (n = 7, *p* < 0.05). Moreover, with cell exposure to ranolazine (10 μM), the peak amplitude of *I*_Na_ decreased from 732 ± 33 to 321 ± 18 pA (n = 7, *p* < 0.05).

The relationship between the MGB concentration and the peak (*I*_Na(T)_) or late (*I*_Na(L)_) component of *I*_Na_ evoked in response to abrupt membrane depolarizing was further analyzed and tested. In this stage of the experiments, each cell was rapidly stepped from −100 to −10 mV and the *I*_Na(T)_ or *I*_Na(L)_ measured at different MGB concentrations was collected and then compared. As Figure 1A,B show, the cumulative addition of MGB in the range of 0.3 to 100 μM results in a concentration-dependent reduction in the amplitude of *I*_Na(T)_ and *I*_Na(L)_. According to a modified Hill equation described in Section 4, the IC_50_ values for the MGB-mediated inhibition of *I*_Na(T)_ and *I*_Na(L)_ were computed to be 19.5 and 7.3 μM, respectively. The data, therefore, reflect that MGB exerts a depressant action on the depolarization activated *I*_Na_ that is concentration-dependently seen in GH_3_ cells, and that the late component of *I*_Na_ (*I*_Na(L)_) decreased to a greater extent than the peak component of the current (*I*_Na(T)_) in its presence.

### 2.2. Kinetic Evaluation of Time-Dependent Block by MGB on I_Na(T)_ in GH_3_ Cells

**Figure 2:**
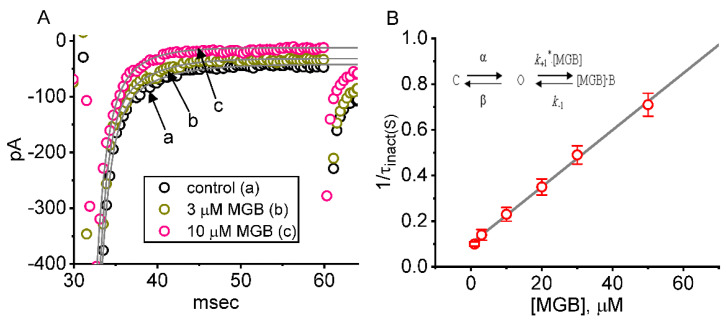
Kinetic assessment of MGB-induced block of *I*_Na_. (**A**) Inactivation time courses of *I*_Na_ evoked by the depolarizing step from −100 to −10 mV for a duration of 30 ms. Each current trajectory in the absence (a), and the presence of 3 μM MGB (b), or 10 μM MGB (c) was well fitted with a least squares criterion by two-exponential decay, i.e., the sum of two exponentials (indicated by the gray smooth line). The values of the fast or slow component (i.e., τ_inact(S)_) in the inactivation time constants of *I*_Na(T)_ obtained in the control period and during exposure to 3 and 10 μM MGB were 1.11, 0.098, and 0.091 ms (fast component), or 4.96, 4.13, and 3.31 ms (slow component), respectively. (**B**) Relationship of the MGB concentration as a function of the slow component in the inactivation rate constant (1/τ_inact(S)_) (mean ± SEM; n = 7 for each point). Of note, the value of 1/τ_inact(S)_ is linearly proportionally to the MBG concentration. Based on the heuristic minimal binding scheme (shown in the Supplementary Information), the value of *k*_+1_^*^ and *k*_−1_ were estimated to be 0.0124 ms^−1^μM^−1^ and 0.102 ms^−1^, respectively; therefore, the *K*_D_ value (*k*_−1_/*k*_+1_^*^, i.e., dissociation constant) turned out to be 8.2 μM, a value which shares a similarity with the IC_50_ value required for its inhibitory effect on *I*_Na(L)_, but smaller than that on *I*_Na(T)_ amplitude.

It was found that increasing the concentration of MGB not only lessened the amplitude of *I*_Na(T),_ but also led to an appreciable raise in the magnitude of current inactivation elicited by rapid membrane depolarization (Figure 2A). We quantitatively measured the inactivation time course of *I*_Na(T)_ at various MGB concentrations. From the first-order binding scheme described in the Supplementary Information, the relationship of 1/τ_inact(S)_ versus the MGB concentration became linear (Figure 2B). The forward (on) and backward (off) rate constants were estimated to be 0.124 ms^−1^μM^−1^ and 0.102 ms^−1^, respectively. Consequently, the apparent dissociation constant (i.e., *K*_D_ = *k*_−1_/*k*_+1_ *) for the binding of MGB to the voltage-gated Na^+^ (Na_V_) channel seen in GH_3_ cells was estimated to yield 8.2 μM. It is of note that the calculated *K*_D_ value has a similarity to the effective IC_50_ needed for MGB to suppress the amplitude of *I*_Na(L)_, although it tends to be measurably smaller than that needed for its reduction in *I*_Na(T)_ amplitude.

### 2.3. Mean Current-Voltage (I-V) Relationship of I_Na(T)_ Caused by MGB

**Figure 3:**
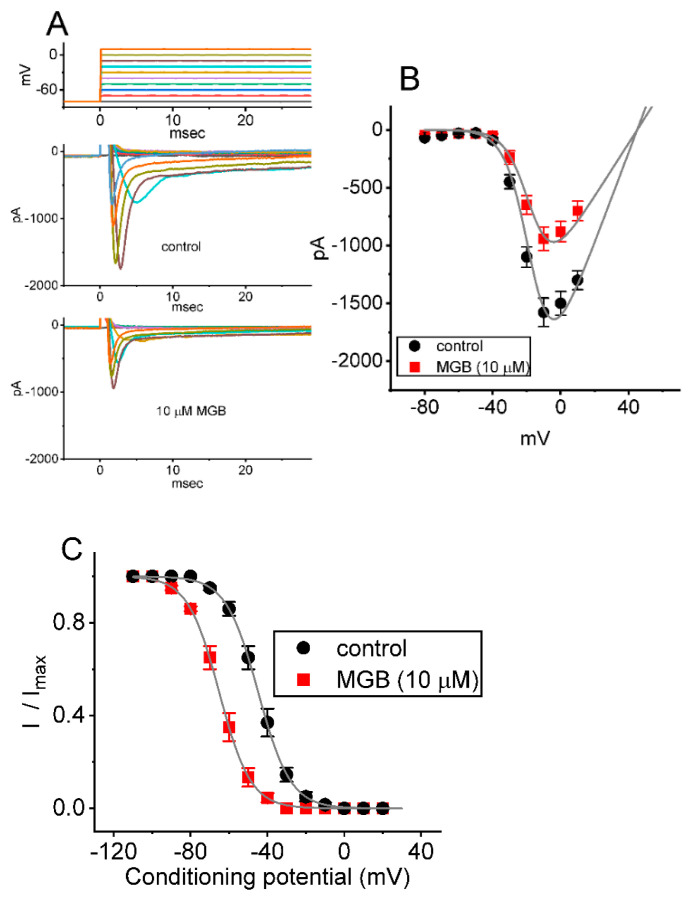
Mean current-voltage (*I-V*) relationship of *I*_Na(T)_ in GH_3_ cells. The preparations made during this series of experiments are the same as those described in Figure 1 and Figure 2. The examined cell was maintained at −80 mV and a series of depolarizing command voltages ranging from −80 to +10 mV in 10 mV steps were applied to it. (**A**) Representative current traces taken in the control period (upper) and during cell exposure to 10 μM MGB. The uppermost part shows the voltage protocol applied. (**B**) Mean *I-V* relationship of *I*_Na(T)_ in the absence (filled black circles) and presence (filled red squares) of 10 μM MGB (mean ± SEM; n = 7 for each point). Current amplitude was measured at the beginning of each depolarizing pulse. Of these, the overall *I-V* relationship of *I*_Na(T)_ (or peak *I*_Na_) seen in GH_3_ cells was unaltered in the presence of MGB. (**C**) Quasi-steady-state inactivation curve of *I*_Na(T)_ in the control (filled black circles) and during exposure to 10 μM MGB (filled red squares) (mean ± SEM; n = 7 for each point). The Boltzmann equations for the *I-V* relation and inactivation curve of *I*_Na(T)_ least squares fitted to generate the smooth lines are described in Materials and Methods.

In the next series of experiments, the *I*_Na_ evoked in response to a series of voltage pulses was examined to test whether the presence of MGB exerts any modifications on *I*_Na(T)_. In these experiments, when the whole-cell configuration was securely established, the tested cell was held at −80 mV, and voltage pulses ranging between −80 and +10 mV in 10-mV steps were applied for a duration of 30 ms. As depicted in Figure 3A,B, cell exposure to MGB at a concentration of 10 μM led to a progressive reduction in *I*_Na(T)_ amplitude, which was concomitantly accompanied by a shortening in the slow component of the inactivation time course of the current (i.e., a decrease in τ_inact(S)_ value). The mean *I-V* relationships of *I*_Na(T)_ acquired in the control period (i.e., in the absence of MGB) and during cell exposure to 10 μM MGB are illustrated in Figure 3B. For example, when the depolarizing command voltage with a range of −80 to −10 mV was applied to the tested cell, the presence of 10 μM MGB evidently lessened the amplitude of *I*_Na(T)_ to 765 ± 88 pA (n = 7, *p* < 0.05) from a control value of 1749 ± 123 pA (n = 7). After the compound was removed, current amplitude returned to 1728 ± 118 pA (n = 7). Furthermore, the reversal potential of peak *I*_Na_ differed between the absence and presence of MGB. The *I-V* curves obtained in the control period (i.e., when MGB was not present) and during exposure to 10 μM MGB were fitted with a Boltzmann function as described in Section 4. In the control test, *G* = 39.1 ± 1.2 nS, *V*_h_ = −16.5 ± 1.9 mV, and *k* = 7.1 ± 0.9 (n = 7), while in the presence of 10 μM MGB, *G* = 22.9 ± 1.1 nS, *V*_h_ = −16.7 ± 1.8 mV, and *k* = 7.2 ± 0.9 (n = 7). These observations indicate that the existence of MGB exerts a depressant action on *I*_Na(T)_ intrinsically in GH_3_ cells, and that the overall *I-V* relationship of *I*_Na(T)_ is unaltered in its presence, although this drug tends to be selective for sustained over peak *I*_Na_ evoked in response to depolarizing command voltages.

In the two-step voltage protocol, the steady-state inactivation curve of *I*_Na(T),_ with or without the application of MGB, was constructed and is hence shown in Figure 3C. In control, *V*_1/2_ = −44.8 ± 1.9 mV, and *q* = 2.9 ± 0.3 *e* (n = 7), while in the presence of 10 μM MGB, *V*_1/2_ = −65.1 ± 2.1 mV, and *q* = 3.0 ± 0.3 *e* (n = 7). Therefore, there was a leftward shift along the voltage axis in the inactivation curve of the current by around 10 mV with no change in the gating charge of the curve.

### 2.4. Effect of MGB on the Recovery from I_Na(T)_ Inactivation Evoked during Varying Interpulse Intervals

**Figure 4:**
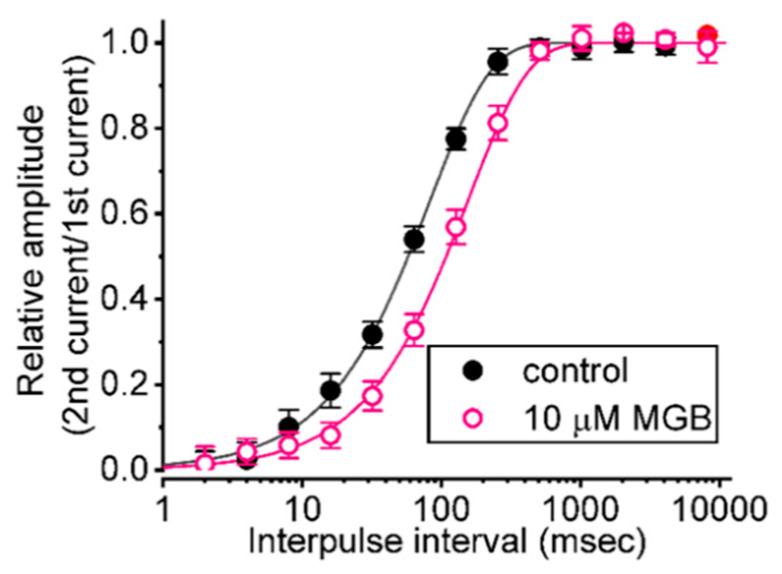
Effect of MGB on the recovery of *I*_Na(T)_ inactivation evoked by varying interpulse intervals with a geometric progression. In these recording experiments, we kept cells bathed in Ca^2+^-free Tyrode’s solution, while the recording pipette was backfilled with K^+^-enriched solution. The examined GH_3_ cells were depolarized from −80 to −10 mV for a duration of 30 ms, and subsequently different interpulse durations with a geometric progression (indicated in the upper part) were delivered to them. The time course of recovery from *I*_Na(T)_ inactivation taken in the absence of (filled black circles) and presence (open pink circles) of 10 μM MGB is illustrated. The relative amplitude of peak *I*_Na_ was measured as a ratio of the second peak amplitude divided by the first peak amplitude peak. The recovery time course (indicated by the smooth line) in the absence of and presence of 10 μM MGB displays an exponential rise as a function of the interpulse interval, with a time constant of 83.2 and 156 ms, respectively. Of note, the *x*-axis is illustrated with a logarithmic scale. Each point is the mean ± SEM (n = 7).

Next, we examined whether the presence of MGB produces any adjustment on the recovery of *I*_Na(T)_ from inactivation, by responding to a two-step voltage protocol in which the interpulse interval increases with a geometric progression (common ratio = 2). In this protocol, a 30 ms step from −80 to −10 mV (prepulse) was firstly applied to the tested cell, and then another 30 ms step to −10 mV (test pulse) was used to inactivate most of the current by varying the duration of the interval between a prepulse and a test pulse. The recovery from current inactivation at the holding potential of −80 mV was then examined at different times with a geometric progression, as presented semi-logarithmically in Figure 4. In the control period (i.e., in the absence of MGB), the peak amplitude of *I*_Na(T)_ nearly completely recovered from inactivation when the interpulse duration reached approximately 1 s. The time constants of recovery from current inactivation acquired in the absence and presence of 10 μM MGB were least squares fitted by a single-exponential function with the values of 83.2 ± 2.1 and 156.1 ± 5.9 ms (n = 7, *p* < 0.05), respectively. The experimental observations indicate that there was a conceivable prolongation in the recovery from inactivation of *I*_Na(T)_ as the cells were exposed to MGB.

### 2.5. Effect of MGB on the Window Component of I_Na_ (I_Na(W)_) Measured from GH_3_ Cells

**Figure 5:**
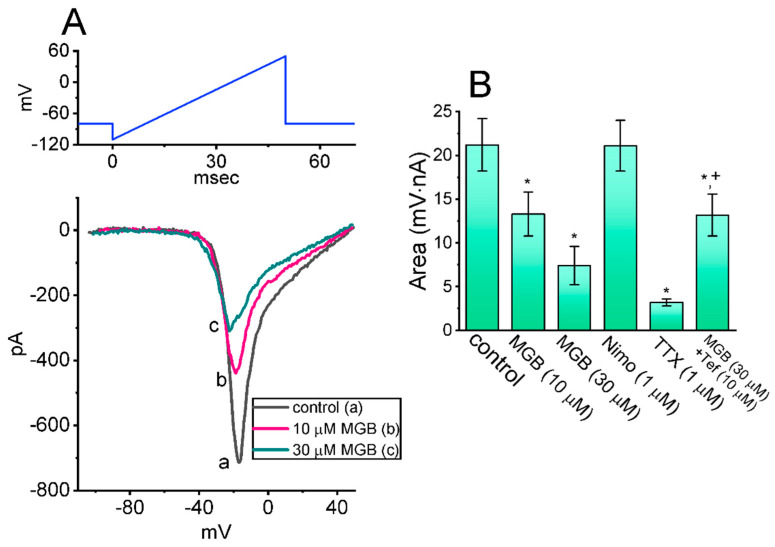
Effect of MGB on window *I*_Na_ (*I*_Na(W)_) elicited by short ascending ramp voltage (V_ramp_). The experiments were conducted with the tested cell voltage-clamped at −80 mV, and the V_ramp_ with a range from −110 to +50 mV was applied for a duration of 50 ms. (**A**) Representative current traces were acquired in the control period (a, black) and during cell exposure to 10 μM MGB (b, pink) or 30 μM MGB (c, green). The voltage protocol used is illustrated in the upper part, and the downward deflection indicates the occurrence of inward current. (**B**) Summary bar graph showing the effect of MGB, nimodipine (Nimo), tetrodotoxin (TTX), and MGB plus tefluthrin (Tef) on the area of *I*_Na(W)_ (mean ± SEM; n = 8). Each area was measured at the voltages ranging between −40 and +40 mV during the upsloping V_ramp_. * This result is significantly different from control (*p* < 0.05) and ^+^ significantly different from MGB (30 μM) alone group (*p* < 0.05).

The presence of instantaneous *I*_Na(W)_ evoked by the ascending (or upsloping) ramp voltage (V_ramp_) was revealed earlier in a variety of excitable cells [37,38,39,40,41]. Next, we explored whether the MGB presence in GH_3_ cells could modify the magnitude of *I*_Na(W)_ activated in response to the rapid ascending V_ramp_. In order to conduct these experiments, the tested cell was voltage-clamped at −80 mV, and we then applied an ascending V_ramp_ from −110 to +50 mV for a duration of 50 ms to evoke *I*_Na(W_) [37]. As disclosed in Figure 5A,B, within one minute of exposing cells to MGB (10 or 30 μM), the amplitude of *I*_Na(W)_ achieved by the 50-ms upsloping V_ramp_ decreased strikingly. For example, the presence of 10 μM MGB strikingly reduced the area of *I*_Na(W)_ measured at the voltage between −40 and +40 mV from 21.2 ± 3.0 to 13.3 ± 2.5 mV·nA (n = 7, *p* < 0.05). After the drug was removed, current amplitude returned to 20.7 ± 3.1 mV·nA (n = 7). The summary bar graph presented in Figure 5B shows that the addition of MGB is effective in decreasing the *I*_Na(W)_ area, and that the subsequent addition of 10 μM tefluthrin (Tef), an insecticide known to be an activator of *I*_Na_ [32,42], overcomes the MGB-mediated reduction in the V_ramp_-induced *I*_Na(W)_ areas. Moreover, the presence of tetrodotoxin (TTX, 1 μM) effectively suppresses the *I*_Na(W)_ area, while that of nimodipine, an inhibitor of L-type Ca^2+^ currents, fails to have any effect on it. Tefluthrin was not shown to be an activator of Ca_V_ channels. The results noted here mean that MGB-mediated suppression of the *I*_Na(W)_ area is not mediated through its inhibitory effect on Ca_V_ channels.

### 2.6. Suppressive Effect of MGB on Resurgent I_Na_ (I_Na(R)_) Seen in GH_3_ Cells

**Figure 6:**
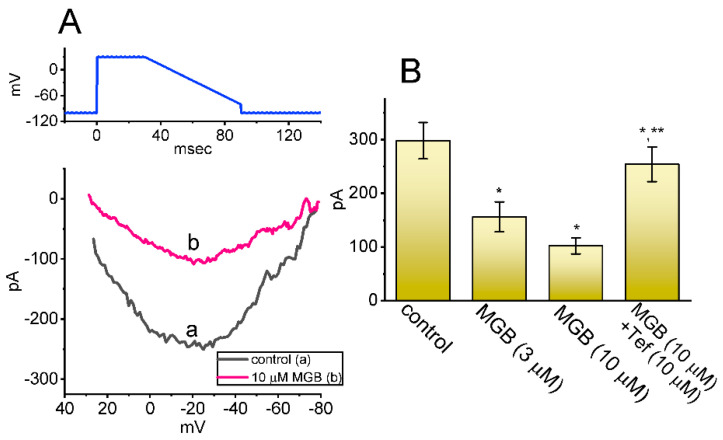
Effect of MGB on resurgent *I*_Na_ (*I*_Na(R)_) evoked by the descending V_ramp_. The tested cell was held at −100 mV and the 30 ms depolarizing pulse at +30 mV was applied. Following the step depolarization, the downsloping V_ramp_ from +30 to −80 mV was delivered to the cell for a duration of 60 ms. (**A**) Representative *I-V* relationships of *I*_Na(R)_ evoked by the descending V_ramp_ in the absence (a, black) and presence (b, pink) of 10 μM MGB. The upper part signifies the voltage protocol used, and the *x*-axis at the lower part is indicated from +40 to −80 mV. (**B**) Summary bar graph showing effects of MGB and MGB plus tefluthrin (Tef) on *I*_Na(R)_ (mean ± SEM; n = 7 for each bar). Current amplitude was measured at the level of −20 mV during the descending V_ramp_. * This result is significantly different from control (*p* < 0.05) and ** significantly different from the MGB (10 μM) alone group (*p* < 0.05).

The *I*_Na(R)_ was identified earlier in GH_3_ cells [32,33], and the magnitude of the current is strongly linked to high-frequency firing observed in Purkinje neurons [43]. In parallel with earlier observations in neurons or endocrine cells [33,44], this type of current is unique in that it is not detectable until the membrane potential is repolarized below 0 mV. In addition to being activated by depolarizing voltage steps rather than by repolarizing voltage steps, *I*_Na(R)_ was observed to activate and decay more slowly than *I*_Na(T)_. The *I*_Na(R)_ is thought to help produce rapid depolarization immediately after an action potential; hence, it is suited either for cells that fire spontaneously at a higher firing rate, or to offer noise modulation in bursting neurons [43,44,45,46]. For these reasons, we additionally investigated whether MGB could exercise any perturbations on such instantaneous current evoked by the descending V_ramp_. As the whole-cell configuration was securely established, the 30 ms depolarizing step from −100 to +30 mV followed by a descending (or repolarizing) V_ramp_ to −80 mV was imposed on the examined cell for a duration of 60 ms. As depicted in Figure 6A,B, the *I*_Na(R)_ amplitude activated by such voltage-clamp protocol evidently reduced during GH_3_ cell exposure to MGB. For example, the presence of MGB at a concentration of 3 or 10 μM MGB led to a decrease of *I*_Na(R)_ at −20 mV from 298 ± 34 pA to 156 ± 28 pA (n = 7, *p* < 0.05) or 102 ± 15 pA (n = 7, *p* < 0.05), respectively. The further addition of Tef (10 μM), still in the presence of 10 μM MGB, restored the *I*_Na(R)_ amplitude at the same level to 254 ± 32 pA (n = 7, *p* < 0.05). However, neither further application of nimodipine (1 μM), nor CdCl_2_ (0.5 mM), exerted any effects on MGB-inhibited *I*_Na(R)_ in the GH_3_ cells (155 ± 27 pA [in the presence of 3 μM MGB plus nimodipine], 156 ± 29 pA [in the presence of 3 μM MGB plus CdCl_2_], versus 156 ± 28 pA [in the presence 3 μM MGB alone]; n = 7, *p* < 0.05). It follows, therefore, that the addition of MGB is capable of suppressing *I*_Na(R)_ in these cells.

### 2.7. Effect of MGB on the Hysteretic Behavior of Persistent Na^+^ Current (I_Na(P)_) Triggered by Isosceles Triangular Ramp Voltage (V_ramp_)

**Figure 7:**
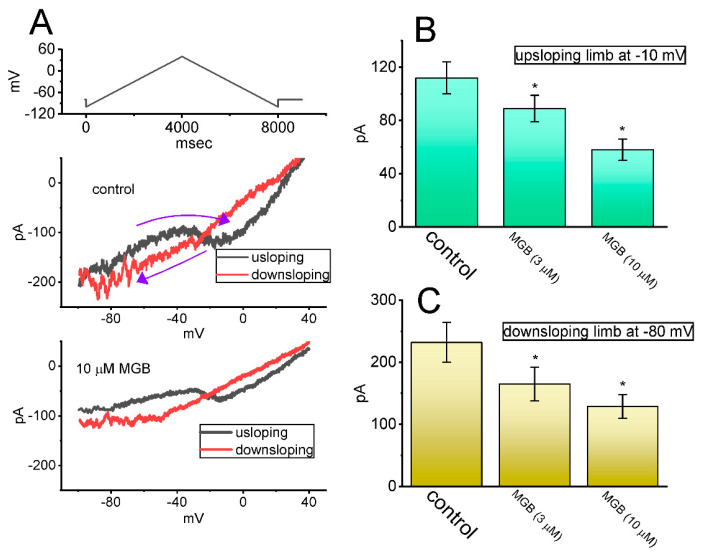
Effect of MGB on persistent *I*_Na_ (*I*_Na(P)_) activated in response to upright isosceles triangular V_ramp_, which was utilized to mimic the depolarizing or repolarizing slopes of bursting patterns in electrically excitable cells. (**A**) Representative current traces activated by isosceles triangular V_ramp_ for a duration of 8 s, or with a ramp speed of ± 75 mV/s (indicated in the uppermost part). The black color in the upper and lower part of (**A**) indicates the current trace activated by the ascending limb of the V_ramp_, while the red color shows trace activated by the V_ramp_’s descending limb. The uppermost part depicts the voltage protocol applied. The purple curved arrow indicates the direction of the current over which time goes during the activation of the triangular ramp pulse. Of note, there is a voltage-dependent hysteresis V_hys_ (i.e., figure of eight configuration) of *I*_Na(P)_ evoked by the isosceles triangular V_ramp_ with or without the MGB (10 μM) addition. In (**B**,**C**), summary bar graphs, respectively, show inhibitory effects of MGB (3 or 10 μM) on the amplitude of *I*_Na(P)_ activated by the upsloping (at −10 mV) and downsloping (at −80 mV) limb of the triangular V_ramp_ (mean ± SEM; n = 7 for each bar). ^*^ This result is significantly different from controls (*p* < 0.05).

Earlier investigations revealed the capability of the V_hys_ strength in *I*_Na(P)_ to affect electrical behaviors in many types of excitable cells [32,35,36,47,48]. Therefore, we attempted to determine whether and how the presence of MGB could modify the *I*_Na(P)_ strength activated in response to a long-lasting upright isosceles triangular V_ramp_. In this series of experiments, during the control period or cell exposure to MGB, we voltage-clamped the examined cell at −80 mV, and an upsloping (ascending) limb from −100 to +50 mV, followed by a downsloping (descending) limb back to −100 mV (i.e., upright isosceles triangular V_ramp_) was applied to it for a duration of 8 s (Figure 7A). As demonstrated earlier [35], under these experimental conditions, the voltage-dependent hysteresis (V_hys_) of *I*_Na(P)_ in response to this triangular V_ramp_ was observed as a striking figure-of-eight (i.e., ∞-shaped) hysteresis in the instantaneous *I-V* relationship of *I*_Na(P)_ (Figure 7A). In other words, there are two distinct loops; that is, the *I*_Na(P)_ amplitude at a high- (i.e., in a counterclockwise direction) threshold loop and at a low- (i.e., in a clockwise direction) threshold loop, activated by the upsloping and downsloping limbs of the upright isosceles triangular V_ramp_. Of notable interest, as shown in Figure 7B,C, during cell exposure to 3 or 10 μM MGB, the strength of current responding to both rising (i.e., high threshold amplitude) and falling (i.e., low threshold amplitude) limbs of isosceles triangular V_ramp_ progressively reduced. For example, on the upright isosceles triangular V_ramp_, the amplitude activated by the ascending ramp at the level of −10 mV in the presence of 3 or 10 μM MGB decreased, respectively, to 89 ± 10 pA (n = 7, *p* < 0.05) or 58 ± 8 pA (n = 7, *p* < 0.05) from a control value of 112 ± 12 pA (n = 7). Likewise, cell exposure to 3 or 10 μM MGB resulted in a measurable decrease in the *I*_Na(P)_ amplitude evoked by the descending ramp at −80 mV to 165 ± 27 pA (n = 7, *p* < 0.05) or 129 ± 19 pA (n = 7, *p* < 0.05), respectively, from a control value of 232 ± 32 pA (n = 7). As such, the findings from this data enabled us to propose an emergence of V_hys_ behavior for *I*_Na(P)_ activation in response to the upright isosceles triangular V_ramp_ in GH_3_ cells, and that the hysteretic strength of the current was measurably reduced by increasing MGB concentration.

### 2.8. MGB-Induced Increase in Cumulative Inhibition of I_Na(T)_ Inactivation

**Figure 8:**
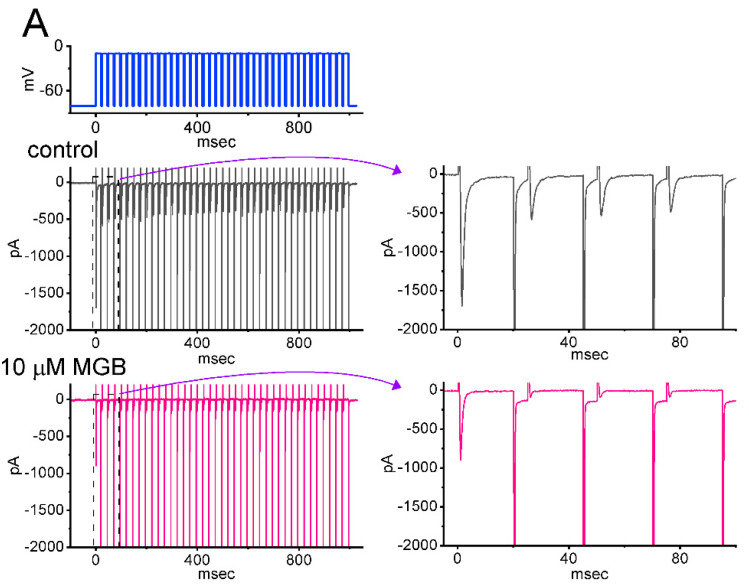

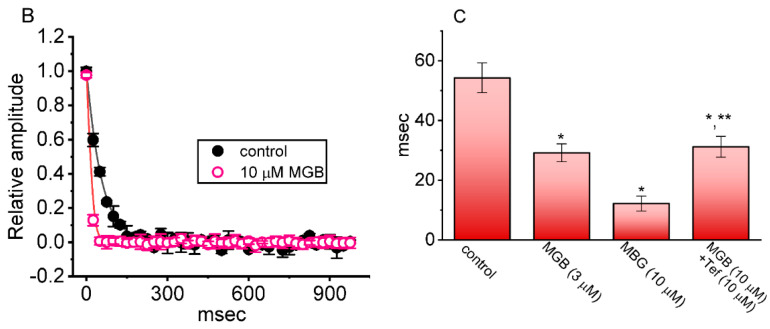
Effect of MGB on *I*_Na(T)_ activated by a train of depolarizing pulses in GH_3_ cells. The train was designed to consist of 40 20 ms pulses (stepped to −10 mV) separated by 5 ms intervals at −80 mV for a duration of 1 s. (**A**) Representative current traces taken in the control period (a, absence of MGB) and during cell exposure to 10 μM MGB. The voltage-clamp protocol is illustrated in the uppermost part. To provide a single *I*_Na_ trace, the right side of (**A**) denotes the expanded records from the dashed box of the left side. (**B**) The relationship of peak *I*_Na_ (*I*_Na(T)_) versus the pulse train duration in the absence (filled black circles) and presence (open pink circles) of 10 μM MGB (mean ± SEM; n = 7 for each point). The continuous smooth lines over which the data points are overlaid are well-fitted by a single exponential. Of note, the presence of MGB can quicken the time course of *I*_Na(T)_ inactivation in response to a train of depolarizing pulses. (**C**) Summary bar graph showing the effect of MGB and MGB plus tefluthrin (Tef) on the time constant of current decay in response to a train of depolarizing command voltage from −80 to −10 mV (mean ± SEM; n = 7 for each bar). Current amplitude was measured at the beginning of each depolarizing pulse. Of note, the presence of MGB produces a significant shortening in the time constant in the decline of peak *I*_Na_ activated by a train of pulses. * Significantly different from control (*p* < 0.05) and ** significantly different from MGB (10 μM) alone group (*p* < 0.05).

*I*_Na(T)_ inactivation was shown to accumulate prior to being activated during repetitive short pulses in previous studies [49,50]. Therefore, additional measurements were taken to study whether the presence of MGB could adjust the inactivation process of the current elicited in a train of depolarizing stimuli. The examined cell was voltage-clamped at −80 mV, and the stimulus protocol, consisting of repetitive depolarization to −10 mV (20 ms in each pulse with a rate of 40 Hz for 1 s), was imposed on it. In keeping with recent observations [50], as depicted in Figure 8A–C, in the control period (i.e., in the absence of MGB), the *I*_Na(T)_ inactivation seen in GH_3_ cells was evoked by a 1 s repetitive depolarization from −80 to −10 mV with an inactivation time constant of 54.3 ± 4.9 ms (n = 7), i.e., showing a sudden current decay with a single-exponential process. It is of interest that during exposure to MGB, at a concentration of either 3 or 10 μM MGB, the exponential time course of *I*_Na_ evoked by the same train of depolarizing pulses shortened to 29.2 ± 3.1 ms (n = 7, *p* < 0.05) or 12.2 ± 2.5 ms (n = 7, *p* < 0.05), respectively, in addition to a reduction in *I*_Na(T)_ amplitude. As cells were continually exposed to 10 μM MGB, the subsequent addition of Tef (10 μM) reversed the MGB-mediated decrease of current decay with a time constant of 31.2 ± 3.5 ms (n = 7, *p* < 0.05). Overall, the results indicate that, apart from the decrease in current magnitude, during cell exposure to MGB the decrease in the decaying of *I*_Na(T)_ elicited by a 1 s train of depolarizing pulses (i.e., accumulative inactivation of the current) can be enhanced in these cells.

## 3. Discussion

The promising findings from this study are that: (a) the existence of MGB depresses *I*_Na_ in a concentration, time-, state-, use-, and hysteresis-dependent manner as identified in GH_3_ cells; (b) this drug resulted in the differential inhibition of *I*_Na(T)_ and *I*_Na(L)_ activated by short step depolarization with the IC_50_ value of 19.5 and 7.3 μM, respectively; (c) the *K*_D_ value of the MGB-induced increase in current inactivation, estimated according to the first-order binding scheme, was 8.2 μM; (d) MGB did not modify the overall *I-V* relationship of *I*_Na(T)_ but the recovery of *I*_Na(T)_ inactivation was prolonged in its presence, while the drug effectively suppressed *I*_Na(W)_ and *I*_Na(R)_ evoked by ascending or descending V_ramp_, respectively; (e) the MGB addition depressed the high- or low-threshold amplitude of *I*_Na(P)_ elicited by the isosceles triangular V_ramp_ at either the upsloping or downsloping limb, respectively; and (f) the cumulative inhibition of *I*_Na_ evoked in response to a train of depolarizing pulses was enhanced in the presence of MGB. Collectively, the present observations show that MGB-mediated changes in the magnitude, gating properties, use-dependence, and hysteretic behavior of *I*_Na_ would potentially modify the functional activities of excitable cells (e.g., GH_3_ cells), presuming that similar in vivo findings are observed.

Perhaps more notable than the issue concerning the magnitude of the MGB-induced reduction in *I*_Na,_ is the observation of the non-linear V_hys_ of *I*_Na(P)_ in the control period (i.e., in the absence of MGB) and during cell exposure to MGB, by use of the upright isosceles triangular V_ramp,_ created through digital-to-analog conversion [47]. During cell exposure to MGB, the peak *I*_Na(P)_ activated by the ascending (upsloping) limb of the triangular V_ramp_ decreased, particularly at the level of −10 mV, while the *I*_Na(P)_ amplitude at the descending (downsloping) limb reduced at the level of −80 mV. In this scenario, the instantaneous figure-of-eight (i.e., infinity-shaped: ∞) residing in the V_hys_ loop that is activated in response to such triangular V_ramp_ appeared indicating that, as the time goes by during activation, there is a counterclockwise direction in the high-threshold loop (i.e., the relationship of current amplitude as a function of membrane potential), followed by a clockwise direction in the low-threshold loop. In other words, there appears to be two types of V_hys_ loop, that is, a high-threshold loop with a peak at −10 mV (i.e., activating at a voltage range near the maximal *I*_Na(T)_ evoked by brief step depolarization), and a low-threshold loop with a peak at −80 mV (i.e., activating at a voltage near the resting potential). The addition of MGB was able to reduce the V_hys_ strength of *I*_Na(P)_. Therefore, findings from these observations reveal that the triangular V_ramp_-induced *I*_Na(P)_ undergoes striking V_hys_ change in the voltage dependence, and that such V_hys_ loops are subjected to attenuation by adding MGB. On the other hand, it needs to be noted that the V_hys_ behavior presented here could be strongly linked to the magnitude of sodium background currents as reported previously [21,48]. Further research should be conducted to understand if MGB-mediated changes in V_hys_ behavior are tightly linked to conformational changes in the voltage sensors of the channel [47].

In this study, the decline of *I*_Na(T)_ during a 40 Hz train of depolarizing pulses (i.e., 20 ms pulses applied from −80 to −10 mV at a rate of 40 Hz for a duration of 1 s) becomes pronounced in the presence of MGB, reflecting that there is use-dependence of *I*_Na(T)_ during repetitive depolarization as recently demonstrated [50], and that cell exposure to MGB would result in a loss-of-function change caused by the altered, quicker inactivation of the current. Therefore, the MGB-mediated decrease of *I*_Na(T)_ is strongly linked to substantial use-dependent facilitation in *I*_Na(T)_ during pulse train stimulation.

An earlier report shows that L-type Ca^2+^ channel activation can up-regulate the mRNAs for two different Na_V_ channels α subunits (Na_V_1.2 and Na_V_1.3) in GH_3_ cells [51]. It is thus postulated that an MGB-induced block of *I*_Na_ seen in GH_3_ cells could be due, in part, to its inhibitory effect on voltage-gated Ca^2+^ currents that are functionally expressed in excitable cells, including GH_3_ cells [52]. However, under our experimental conditions, the voltage-activated inward currents shown herein were either sensitive to stimulation by Tef or subjected to inhibition by TTX and ranolazine. Tef and ranolazine have been reported to be activators or inhibitors of *I*_Na_, respectively [24,32]. In the continued presence of MGB, further addition of Tef could reverse its suppression of *I*_Na(W)_ or *I*_Na(R)_. It has been previously demonstrated that *I*_Na(W)_ and *I*_Na(R)_ are responsible for the electrical firing of excitable cells [38,40,43]. Moreover, neither the presence of nimodipine nor CdCl_2_ effectively suppressed such inward currents in GH_3_ cells. Therefore, it is conceivable that the *I*_Na_ (*I*_Na(L)_, *I*_Na(W)_, *I*_Na(R)_ and *I*_Na(P)_) in GH_3_ cells is susceptible to being inhibited by MGB, and that its block on *I*_Na(L)_ is actually larger than its block on *I*_Na(T)_. Moreover, the reduction in *I*_Na(L)_ caused by a blocker such as ranolazine can lead to a diminution in Ca^2+^ overload by increasing the driving force for Ca^2+^ extrusion through the Na^+^-Ca^2+^ exchanging process that is functioning in reverse mode (i.e., in a mechanism that operates to extrude Ca^2+^, in exchange for the influx of Na^+^) [53,54]. The mRNA transcripts for the α-subunits of Na_V_1.1, Na_V_1.2, and Na_V_1.6 were demonstrated to be present in GH_3_ cells [55]. However, it remains to be determined to what extent MGB can modify *I*_Na_ (e.g., Na_V_1.7 and Na_V_1.8) in dorsal root ganglion neurons, even though such an action could be important for explaining its analgesic potential.

It needs to be emphasized that ranolazine, an inhibitor of *I*_Na(L)_, has been demonstrated to be of benefit for peripheral or diabetic neuropathy [56,57,58,59]. This drug has also been revealed to have modifications on changes in peripheral nerve excitability [24,57,58,60,61,62]. As a corollary, it is tempting to anticipate that MGB-mediated alleviation of painful sensation could be partly, if not entirely, attributable to the inhibitory action on different types of *I*_Na_. While the detailed ionic mechanism of its inhibitory action on the Na_V_ channel is not entirely clear, the MGB molecule may have the propensity to exert a higher effect on the open/inactivated state than on the resting (closed) state residing in the channel, thereby de-stabilizing the open conformation.

Earlier pharmacokinetic studies show that a peak plasma concentration of MGB reached 1000 ng/mL (4.8 μM) one hour after oral administration of 75 mg [63,64]. The effects of MGB on membrane excitability could be likewise dependent on various factors, such as the MGB concentration used, various firing patterns of action potentials [40,43,45], the level of pre-existing resting potential, and in any combinations. It has been noted that some isoforms of the Na_V_ channel α-subunit were engaged in inflammatory pain states, and they were functionally expressed by somatosensory primary afferent neurons, but not by skeletal or cardiovascular muscle [24,65]. We hitherto demonstrated that the MGB action on excitable membranes is not solely explained by its aberrant use as a blocker on α_2_δ subunit of Ca_V_ channels [1,2]. The activity of Na_V_ channels in excitable cells may noticeably confer the susceptibility to perturbations by MGB or its structurally similar compounds.

## 4. Materials and Methods

### 4.1. Chemicals, Drugs and Solutions Used in This Work

Mirogabalin (MGB, Tarlige^®^, DS-5565, (1R,5S,6S)-6-(aminomethyl)-3-ethyl-bicyclo [3.2.0] hept-3-ene-6-acetic acid, C_12_H_19_NO_2_, CAS No.: 1138245-13-2, purity: ≥98%), was from Cayman Chemical (Genechain Industrial, Kaohsiung, Taiwan), while nimodipine, ranolazine, tefluthrin (Tef), tetraethylammonium chloride (TEA), and tetrodotoxin (TTX) were from Sigma (Merck, Taipei, Taiwan). Unless specified otherwise, cell culture media (e.g., Ham’s F-12 medium), horse serum, fetal calf serum, L-glutamine, and trypsin/EDTA were supplied by HyClone^TM^ (Merck, Kenilworth, NJ, USA). All other chemicals, such as CdCl_2_, CsCl, CsOH, HEPES, and aspartic acid were of the best available quality, mostly at analytical grades.

The ionic composition of extracellular solution (i.e., HEPES-buffered normal Tyrode’s solution) was as follows (in mM): NaCl 136.5, KCl 5.4, CaCl_2_ 1.8, MgCl_2_ 0.53, glucose 5.5, and HEPES 5.5 (pH 7.4 adjusted with NaOH). To record K^+^ currents, the electrode was filled up the internal solution containing (in mM): K-aspartate 130, KCl 20, KH_2_PO_4_ 1, MgCl_2_ 1, EGTA 0.1, Na_2_ATP 3, Na_2_GTP 0.1, and HEPES 5 (pH 7.2 adjusted with KOH). To measure Na^+^ currents, we replaced K^+^ ions inside the pipette solution with equimolar Cs^+^ ions, and the pH was titrated to 7.2 by adding CsOH. All solutions were prepared using deionized water which was produced by a Milli-Q water purification system (Merck, Kenilworth, NJ, USA).

### 4.2. Cell Culture

GH_3_, a clonal cell line derived from a rat prolactin-secreting pituitary tumor, was acquired from the Bioresources Collection and Research Center (Hsinchu, Taiwan), and the detailed methodology was described earlier [66]. Briefly, cells were maintained in Ham’s F-12 medium (HyClone^TM^, Logan, UT, USA) with 15% (*v/v*) heat-inactivated horse serum, 2.5% (*v/v*) fetal calf serum, and 2 mM L-glutamine (HyClone^TM^, Logan, UT, USA) in a humidified atmosphere of CO_2_/air (1:19). The culture medium was changed every 2 to 3 days, and cells were passaged when they reached confluence. Cell viability was evaluated using WST-1 assay (Roche Diagnostics, Taipei, Taiwan). To promote differentiation, cells were transferred to a serum-free, Ca^2+^-free medium. Under these experimental conditions, cells remained 80 to 90% viable for at least 2 weeks. The measurements were conducted 5 or 6 days after cells were grown to 60–80% confluence.

### 4.3. Electrophysiological Measurements

Before the experiments, we gently dispersed cells with a 1% trypsin/EDTA solution, and an aliquot of cell suspension was directly placed in a recording chamber attached to the fixed-stage of a DM-IL inverted microscope (Leica; Highrise Instrument, Taichung, Taiwan). Cells were immersed at room temperature (20–25 °C) in normal Tyrode’s solution containing 1.8 mM CaCl_2_. The electrodes that we used were fabricated from Kimax-51 capillaries (Merck, Taipei, Taiwan) using a PP-83 vertical puller (Narishige; Taiwan Instrument, Tainan, Taiwan), and their tips were thereafter fire-polished with an MF-83 microforge (Narishige; Taiwan Instrument, Tainan, Taiwan). As the electrodes were filled with the different internal solutions described above, their resistance was measured to range between 3 and 5 MΩ, for the purpose of avoiding excessive damage to the cell. Patch-clamp recordings were carried out in whole-cell configuration using either an RK-400 (Bio-Logic, Claix, France) or an Axopatch-200B amplifier (Molecular Devices; Bestgen Biotech, New Taipei City, Taiwan), as described elsewhere [31,37,52,67]. Whole-cell recording was achieved by rupturing the patch of membrane isolated with GΩ sealing by the patch pipet, which brings the cell interior into contact with the pipet interior.

### 4.4. Data Recordings and Analyses

The signals were simultaneously monitored with a digital oscilloscope (Gould, Chandler, AZ, USA) and a liquid crystal display projector (ViewSonic, Walnut, CA, USA). The data were stored online in a Sony VAIO CS series laptop computer (VGN-CS110E; Tainan, Taiwan), equipped with 1440A digitizer (Molecular Devices). During the measurements with analog-to-digital and digital-to-analog conversion, the latter device was controlled by pCLAMP 10.6 software (Molecular Devices) run on Microsoft Windows 7 (Redmond, WA, USA). The laptop computer used was put on the top of an adjustable Cookskin stand (Ningbo, Zhejiang, China) for convenient manipulation during the experiments. To ensure digitalization, in some sets of measurements, we collected current signals by PowerLab 2/26 acquisition system (ADInstruments; Kuo Yang, Taipei, Taiwan).

To better evaluate the concentration–response curve of MGB-mediated inhibition on the peak (transient, *I*_Na(T)_) and sustained (late, *I*_Na(L)_) components of *I*_Na_, *I*_Na_ were evoked by 30 ms depolarizing pulse to −10 mV from a holding potential of −100 mV, and current amplitudes taken with or without the application of different MGB concentrations (0.3–100 μM) were measured at the start (*I*_Na(T)_) and end pulse (*I*_Na(L)_) of the depolarizing pulse. The concentration required to inhibit 50% of current amplitude was determined according to the three-parameter logistic model (i.e., a modified form of sigmoidal Hill equation) by use of goodness-of-fit assessments:

$$Relative\;amplitude=\frac{{\left[MGB\right]}^{-n_{H}}\times \left(1-a\right)}{{\left[MGB\right]}^{-n_{H}}+IC_{50}^{-n_{H}}}+a$$

where, *n_H_* = the Hill coefficient (i.e., the number bound per side); *IC_50_* = the concentration required for a 50% inhibition); and [*MGB*] = the MGB concentration. Maximal inhibition (i.e., *1 − a*) was approximated in this equation.

The *I-V* relationship of *I*_Na(T)_ with or without addition of MGB was constructed and thereafter fitted with a Boltzmann function given by:

$$\frac{I}{I_{max}}=\frac{G}{1+exp\left[-\left(V-V_{h}\right)/k\right]}\times \left(V-E_{rev}\right)$$

where *V* is the membrane potential in mV, *E*_rev_ the reversal potential of *I*_Na_ (fixed at +45 mV), *G* the Na^+^ conductance in nS, and *I* the current in pA, while *k* and *V*_h_ are the gating parameters.

To evaluate the steady-state inactivation of *I*_Na(T)_ with or without the existence of MGB, a two-step voltage protocol was created and delivered to the tested cells. A 30 ms conditioning pulse to various membrane potentials preceded the test pulse (30 ms in duration) to −10 mV from a holding potential of −80 mV. The relationship between the normalized amplitude of *I*_Na(T)_ and the conditioning potentials was appropriately fitted with another Boltzmann function of the following form:

$$\frac{I}{I_{max}}=\frac{1}{1=exp\left[\left(V-V_{\frac{1}{2}}\right)qF/RT\right]}$$

where *I*_max_ is the maximal amplitude of *I*_Na(T),_ *V*_1/2_ the voltage at which half-maximal inhibition occurs, *q* the apparent gating charge of the inactivation curve, *F* Faraday’s constant, *R* the universal gas constant, and *T* the absolute temperature.

The kinetic evaluation of the MGB-mediated effect on *I*_Na(T)_ inactivation is provided in the Supplementary Information.

### 4.5. Curve-Fitting Procedures and Statistical Analyses

Linear or nonlinear curve fitting to experimental data sets in this study was undertaken with the interactive least squares procedure by using different maneuvers, such as Microsoft Excel^®^-embedded “Solver” (Microsoft, Redmond, WA, USA) and OriginPro^®^ 2021 program (OriginLab; Scientific Formosa, Kaohsiung, Taiwan). The averaged results are presented as the mean ± standard error of the mean (SEM) with the sizes of observations (n) indicating the cell numbers from which samples were taken. The paired or unpaired Student’s *t*-tests between the two groups were applied. When the differences among different groups were encountered, we performed either analysis of variance (ANOVA)-1 or ANOVA-2 with or without repeated measures followed by post-hoc Fisher’s least significant difference test. Statistical analyses were made using the SPSS 20 package (IBM; Tainan, Taiwan). Statistical significance (indicated with * in the figures) was determined at a *p* value of < 0.05.

## Data Availability

The original data is available upon reasonable request to the corresponding author.

## Supplementary Materials

The following supporting information can be downloaded at: https://www.mdpi.com/article/10.3390/ijms23073845/s1.

## Abbreviations

Ca_V_ channel	voltage-gated Ca^2+^ channel
*I-V*	current versus voltage
IC_50_	concentration required for 50% inhibition
*I*_Na_	voltage-gated Na^+^ current
*I*_Na(L)_	late Na^+^ current
*I*_Na(P)_	persistent Na^+^ current
*I*_Na(R)_	resurgent Na^+^ current
*I*_Na(T)_	transient Na^+^ current
*I*_Na(W)_	window Na^+^ current
*K*_D_	dissociation constant
MGB	mirogabalin (Tarlige^®^, 1R,5S,6S)-6-(aminomethyl)-3-ethyl-bicyclo [3.2.0] hept-3-ene-6-acetic acid)
N_aV_ channel	voltage-gated Na^+^ channel
SEM	standard error of the mean
TEA	tetraethylammonium chloride
Tef	tefluthrin
TTX	tetrodotoxin
V_hys_	voltage-dependent hysteresis
V_ramp_	ramp voltage
