# Supplementary material for: The Evidence for Effective Inhibition of INa Produced by Mirogabalin ((1R,5S,6S)-6-(aminomethyl)-3-ethyl-bicyclo [3.2.0] hept-3-ene-6-acetic acid), a Known Blocker of CaV Channels
Source: Int J Mol Sci. 2022 Mar 31;23(7):3845. doi: 10.3390/ijms23073845 (PMC8998350; doi:10.3390/ijms23073845)
Supplement: Supplementary file 1 [file ijms-23-03845-s001.zip › ijms-1620300-supplementary.pdf]

Supplementary Information

Kinetic evaluation for MGB-induced block of $I_{Na(T)}$ evoked by short step depolarization in pituitary GH_3 cells

During cell exposure to MGB, apart from the reduction in peak I_{Na} (i.e., $I_{Na(T)}$), the inactivation rate of the current elicited by brief depolarizing pulse from -100 to -10 mV was noticed to become elevated. Hence, we extended our investigations to evaluate the inactivation kinetics of MGB-mediated suppression of $I_{Na(T)}$ in situations where GH_3 cells were exposed to different MGB concentrations. The concentration dependence of I_{Na} inactivation rate (i.e., $1/\tau_{inact(S)}$) caused by adding this drug was constructed and is hence illustrated in **Figure 2B**. The observations indicated that its effects on $I_{Na(T)}$ resulted in a concentration-dependent increase in the slow component in rate of current inactivation, since the inactivation trajectories of the current were well fitted at various MGB concentrations to two-exponential process. As a result, the inhibitory effect on $I_{Na(T)}$ inherently in GH_3 cells can be reasonably explained by a state-dependent blocking mechanism through which the MGB molecule can predominantly bind to the open or open-inactivated state residing in the Na_V channels. The first-order binding scheme for this perspective is thus simplified as follow.

$$C \underset{\beta}{\overset{\alpha}{\rightleftharpoons}} O \underset{k_{-1}}{\overset{k_{+1}^{*}\cdot[MGB]}{\rightleftharpoons}} [MGB]\cdot B$$

where α and β are called the rate constants for the opening and closing of the Na_V channel, respectively; k_{+1}^{*} and k_{-1} are those for blocking (i.e., depending on the MGB concentration) or unblocking by adding MGB; [MGB] is the MGB concentration used. C, O, and [MGB]·B indicate the closed (or resting), open, and blocked states of the Na_V channel, respectively.

The blocking (forward, k_{+1}^{*}) and unblocking (backward, k_{-1}) rate constants were estimated based on the slow component of the inactivation time constant ($\tau_{inact(S)}$) of $I_{Na(T)}$ during cell exposure to different MGB concentrations (**Figure 2B**). The relationship can be satisfied by

$$1/\tau_{inact(S)} = k_{+1}^{*} \times [MGB] + k_{-1}$$

where k_{+1}^{*} and k_{-1} were respectively derived from the slope (i.e., $\Delta(1/\tau_{inact(S)})/\Delta([MGB])$ and from the y-axis intercept at [MGB]=0 (i.e., the point at which the line crosses the y-axis) of the linear regression at which the reciprocal time constants of the current (i.e., $1/\tau_{inact(S)}$) versus the MGB concentration was interpolated. The blocking and unblocking rate constants were therefore estimated to yield 0.124 $msec^{-1}\mu M^{-1}$ and 0.102 $msec^{-1}$, respectively. According to the evolving rate constants, dividing k_{-1} by k_{+1}^{*} gave a dissociation constant (K_D) of 8.2 μM during exposure to MGB, a value that was noticed to be close to effective IC_{50} (7.3 μM) of the drug needed for inhibition of late I_{Na} (**Figure 1B**).
